# PRIORITIES TO IMPROVE DEMENTIA CAREGIVING IN CHINESE IMMIGRANT FAMILIES: REPORT OF A CAREGIVER WORKSHOP

**DOI:** 10.1093/geroni/igae098.3627

**Published:** 2024-12-31

**Authors:** Kris (Pui Kwan) Ma, Anne Saw, Joyce Cheng

**Affiliations:** University of Washington, Seattle, Washington, United States; DePaul University, Chicago, Illinois, United States; Chinese Community Health Resource Center, San Francisco, California, United States

## Abstract

Linguistically diverse caregivers of people with dementia represent a growing but under-recognized segment of the dementia care workforce in the U.S. There is a continued shortage of culturally and linguistically responsive dementia caregiving interventions for older U.S. Chinese immigrants with limited English proficiency. To address the unmet needs of Chinese-speaking caregivers, integrating caregivers’ input is vital to studies to ensure that research aligns with their experiences. This was a focus group study of 10 Chinese-speaking caregivers (mean age 73.7, eight women) conducted during a dementia caregiving workshop, in partnership with a community health resource center in California. During the in-language workshop, caregivers identified their needs in surveys, then discussed with one another in the focus group. Consensus was built to establish priority areas of research and clinical practice to address caregivers’ experiences and needs. We used descriptive and thematic analyses to summarize caregivers’ priorities. Caregiver-identified priorities included information and practical support in a) management of behavioral and psychological symptoms of dementia (BPSD), b) communication with care recipients and other family members, c) care for caregivers’ physical and mental health, and d) assistance to connect to in-language community resources. We identified ways in which these priorities can inform dementia caregiving research and practice for Chinese-speaking caregivers and immigrant families in the U.S. It is recommended that future research and interventions for Chinese-speaking immigrant caregivers in the U.S. prioritize BPSD management skills training, adopt a family-centered approach and include family outcomes, and address service barriers via community-clinic partnerships and outreach.

